# Chronic inflammation, cancer development and immunotherapy

**DOI:** 10.3389/fphar.2022.1040163

**Published:** 2022-10-14

**Authors:** Yalei Wen, Yingjie Zhu, Caishi Zhang, Xiao Yang, Yuchen Gao, Mei Li, Hongyan Yang, Tongzheng Liu, Hui Tang

**Affiliations:** ^1^ College of Pharmacy/International Cooperative Laboratory of Traditional Chinese Medicine Modernization and Innovative Drug Development of Ministry of Education (MOE) of China, Jinan University, Guangzhou, China; ^2^ Department of Central Laboratory, The First Affiliated Hospital of Jinan University, Guangzhou, China; ^3^ Department of Clinical Laboratory, The Fifth Affiliated Hospital of Jinan University (Heyuan Shenhe People’s Hospital), Heyuan, China

**Keywords:** cancer development, chronic inflammation, tumor microenvironment (TME), immunotherapies, metastasis, therapeutic resistance

## Abstract

Chronic inflammation plays a pivotal role in cancer development. Cancer cells interact with adjacent cellular components (pro-inflammatory cells, intrinsic immune cells, stromal cells, etc.) and non-cellular components to form the inflammatory tumor microenvironment (TME). Interleukin 6 (IL-6), macrophage migration inhibitory factor (MIF), immune checkpoint factors and other pro-inflammatory cytokines produced by intrinsic immune cells in TME are the main mediators of intercellular communication in TME, which link chronic inflammation to cancer by stimulating different oncogenic signaling pathways and improving immune escape to promote cancer development. In parallel, the ability of monocytes, T regulatory cells (Tregs) and B regulatory cells (Bregs) to perform homeostatic tolerogenic functions is hijacked by cancer cells, leading to local or systemic immunosuppression. Standard treatments for advanced malignancies such as chemotherapy and radiotherapy have improved in the last decades. However, clinical outcomes of certain malignant cancers are not satisfactory due to drug resistance and side effects. The clinical application of immune checkpoint therapy (ICT) has brought hope to cancer treatment, although therapeutic efficacy are still limited due to the immunosuppressive microenvironment. Emerging evidences reveal that ideal therapies including clearance of tumor cells, disruption of tumor-induced immunosuppression by targeting suppressive TME as well as reactivation of anti-tumor T cells by ICT. Here, we review the impacts of the major pro-inflammatory cells, mediators and their downstream signaling molecules in TME on cancer development. We also discuss the application of targeting important components in the TME in the clinical management of cancer.

## 1 Introduction

A functional linking between cancer and chronic inflammation has long been noted. In 1863, Virchow first described leukocyte infiltration within tumors and hypothesized that cancer originated from the sites of chronic inflammation (Korniluk et al., 2017). Tissue damages and the consequent chronic inflammation caused by certain types of irritants enhance cell proliferation, which might potentiate neoplastic risk when orchestrated with other risk factors of cancer (Taniguchi and Karin 2018). Currently, tumor-associated inflammation is considered as the seventh biological feature of cancer (Colotta et al., 2009). Emerging evidences demonstrate that tumor-associated inflammation could affect almost every stage of cancer: cancer development, metastasis, drug resistance and cancer recurrence by inducing genomic instability, driving self-renewal of cancer stem-like cells (CSCs) and angiogenesis (Atsumi et al., 2014). In other words, chronic inflammation is showed to interfere with tissue homeostasis through the accumulation of various immune cells, thereby evolving a more lethal clonal form of tumor (Whiteside 2008). In addition, various pro-inflammatory mediators from immune cells or cancer cells can promote cancer development and drug resistance (Fares et al., 2020). Therefore, it is crucial to investigate how chronic inflammation is associated with cancer development and develop new-targeted therapies.

In the inflammatory tumor microenvironment (TME), innate immune cells such as monocytes and adaptive immune cells (e.g., T lymphocytes) directly contact or produce a variety of inflammatory mediators [e.g., tumor necrosis factor (TNF), IL-6] in response to aberrant signals from the tumor (Wang et al., 2019; Sahai et al., 2020). These inflammatory mediators further activate multiple pivotal signaling pathways including Janus kinase/signal transducers and activators of transcription (JAK/STAT) and nuclear factor kappa B (NF-κB), which restricts the efficacy of chemotherapy by promoting cancer development and suppressing anti-tumor immunity (Liu and Cao 2016). Thus, many studies have focused on improving therapeutic efficacy by targeting and remodeling TME. This review discusses how chronic inflammation affects the following aspects of malignancy: cancer development, immune escape, and drug resistance. We also review research advances in TME characterization and immune checkpoint. Finally, we summarize the application of targeting TME, immune checkpoint therapy (ICT) and combination therapy in the clinical management of cancers.

## 2 Influence of inflammation on cancer development

### 2.1 Chronic inflammation affects cancer development

**FIGURE 1:**
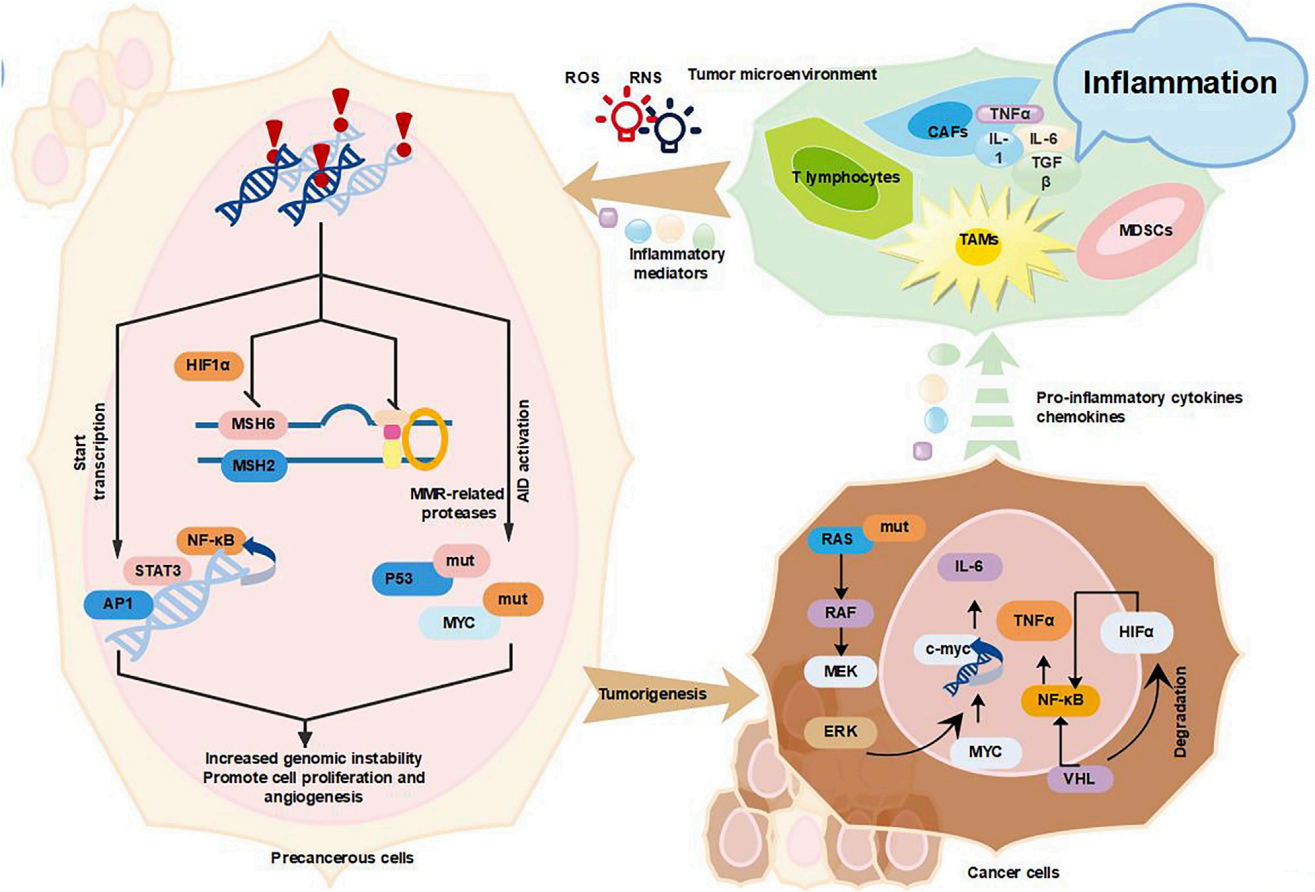
Inflammation promotes cancer development. Tumor-associated inflammation causes excessive release of large amounts of inflammatory factors (TNF-α, IL-6, TGF-β, and IL-1β), ROS and RNS from activated inflammatory cells (mainly TAMs, CAFs, MDSCs, and T lymphocytes), which in turn activate AID and mutate key cancer-related genes *P53* and *MYC* (Elinav et al., 2013, Colotta et al. 2009, Kidane et al. 2014); inflammation increases genomic instability by suppressing MMR, increasing genomic instability, which leads to the accumulation of random genetic alterations (Guerra and Otton 2011; Shimizu et al., 2012; Chaudhry et al., 2013); inflammation will also promote the proliferation and survival of tumor progenitor cells by inducing the expression of growth factors and cytokines NF-κB, STAT3, and AP1, and the above three mechanisms together promote tumor formation (Yu, Ling and Wang 2018). In addition, various types of oncogenes and tumor suppressor genes such as (*KRAS*, *MYC,* and *VHL*) can also promote tumor progression through the recruitment and activation of inflammatory cells (MDSCs and TAMs) (Yu et al., 2018).

Cancer development requires intrinsic and extrinsic factors such as genomic instability, abnormalities in proliferation and senescence, reprogramming energy metabolism, evasion of immune destruction, epithelial mesenchymal transition (EMT). Only 5%–10% of cancer cases driven by germline mutations, while the rest of cancers are caused by acquired factors such as chronic infections, dietary factors, obesity, inhalation of pollutants, smoking and autoimmune related factors (Table 1) (Hibino et al., 2021). These carcinogenic factors have common features of disrupting tissue homeostasis and producing a continuous protective response---chronic inflammation. The environment rich in inflammatory cells, cytokines and DNA damage leads to the various epigenetic changes, and accumulation of mutations in adjacent epithelial cells produces procarcinogenic effects such as immunosuppression and angiogenesis, thereby promoting sustained cell proliferation and tumor initiation (Figure 1) (Greten and Grivennikov 2019a).

**TABLE 1:** Chronic inflammation and tumor-related diseases.

Chronic inflammation that causes insults or pathological conditions	Associated malignancy
Microbial-induced Th17 response-driven pancreatitis; Trypsinogen mutation-associated pancreatitis and alcohol-associated pancreatitis	Pancreatic cancer Mumm and Oft (2008)
Gut bacteria; Lung microbiome; Inflammatory bowel disease	Colorectal cancer Kanmani, Suganya and Kim (2020)
Alcoholic and non-alcoholic fatty liver, Hepatitis B and C	Liver cancer Haga et al. (2015)
Gastritis caused by *helicobacter pylori*	Stomach cancer Carrasco and Corvalan (2013)
Bladder inflammation caused by schistosomiasis	Bladder Cancer Santos et al. (2021)
UV irradiation-associated skin inflammation	Melanoma Landskron et al. (2014)
Obesity and diabetes	Breast cancer Rose, Gracheck and Vona-Davis (2015)
Hyperthermia; Asbestos; Reflux esophagitis	Esophageal cancer Loria, Barrios and Zanetti (2009)

Persistent chronic inflammation activates pro-oncogenes and/or inactivate suppressor genes, cause genomic instability and induce tumors by promoting tumor cell proliferation and survival (Jiang et al., 2019a). Chronic inflammation is a step-by-step process that involves damage, repair and regression (Chen et al., 2018). Inflammatory cells such as lymphocytes, tumor-associated macrophages (TAMs), myeloid-derived suppressor cells (MDSCs) and fibroblasts, are recruited to inflammatory sites where they target pathogens and release cytokines to further elicit inflammation, immune responses and cell proliferation (Li et al., 2019). Excess cytokines, chemokines, growth factors and proteases can stimulate tissue regeneration and reconstruct the extracellular matrix (ECM) (Reuter et al., 2010; Wang et al., 2019; Zhou et al., 2021a). These factors and mediators can also cause cellular DNA damage and impair DNA damage repair response, thus causing genomic instability and contributing to transformation and tumorigenesis. For instance, reactive oxygen species (ROS) produced in monocyte or cytokines IL-1β, transforming growth factor beta (TGF-β) and TNF were demonstrated to cause abnormal expression of activation-induced cytidine deaminase (*AID*), a key B cell enzyme mediating somatic hypermutation, class switching and antibody diversification. *AID* can induces *TP53* and *MYC* mutations in normal cells, thereby promoting cancer development (Colotta et al., 2009). In addition, chronic inflammation may directly disrupt the cell cycle checkpoints and inhibit DNA damage repair responses, thereby leading to the accumulation of random genetic alterations and genomic instability (Colotta et al., 2009; Elinav et al., 2013; Kidane et al., 2014). Chronic inflammation is also reported to induce *P53*-mediated inactivation of genomic surveillance and acceleration of mutations in cancer cells (Mantovani et al., 2008). In addition, chronic inflammation is showed to inhibit mismatch repair (MMR) pathway through various mechanisms and increase the DNA replication error rates of the genome. Inflammatory mediators [prostaglandin E2 (PGE2), ROS, TNF, and IL-1β] induce hypoxia-inducible factor 1α (HIF1α) to downgrade mutS homolog 2 (MSH2) and MSH6 expression by displacing *c-MYC* from the MMR gene (Elinav et al., 2013). Altogether, it is shown that inflammation affects genomic instability and promotes the cancer development.

Conversely, cancer-associated genes are related with the recruitment and activation of pro-inflammatory cells such as bone marrow monocytes to coordinately alter the cancer transcriptional program (Greten and Grivennikov 2019b). For example, the oncogenic gene *RAS* drives multiple inflammatory cytokines (IL-1, IL-6, IL-8, IL-23, etc.)/chemokines [chemokine (C-X-C motif) ligand 1 (CXCL1), CXCL2, CXCL5, CXCL8, and C-X-C motif chemokine receptor 2 (CXCR2), etc.] and signaling pathways [STAT3, NF-κB, mitogen-activated protein kinase (MAPK)] that promote tumor progression (Hamarsheh et al., 2020). Oncogenic *RAS*-induced secretion of IL-6 can link chronic inflammation and cancer. IL-6-mediated activation of the JAK/STAT3 signaling pathway by autocrine or paracrine means is required for malignant tumor proliferation (Browning et al., 2018). The activated cancer associated fibroblasts (CAFs) promote the immune checkpoint molecule expression and ECM remodeling, thereby suppressing the tumor immune response and promoting tumor development (Bansod, Dodhiawala and Lim 2021). Another important cancer-related gene is *MYC* (Beroukhim et al., 2010). *MYC* can directly regulate tissue remodeling, angiogenesis and chronic inflammation to exert oncogenic activity (Mantovani et al., 2008). In a reversibly switchable mouse transgenic model of *MYC*-dependent β-cell carcinogenesis, the dramatic activation of *MYC* in β-cells triggers the release of IL-1β, induces the proliferation of adjacent endothelial cells, thereby leading to the generation of new complex vessels (Shchors et al., 2006). Activation of *MYC* also promotes the recruitment of mast cells to tumor sites, which is required for tumor angiogenesis and macroscopic expansion (Theoharides 2008).

Tumor suppressor proteins can also suppress cancer development by inhibiting the production of inflammatory mediators. Von Hippel-Lindau (VHL) largely exerts tumor suppression by targeting HIF1α and HIF2α to oxygen-dependent ubiquitination and proteasome-dependent degradation (Joshi et al., 2014). The inactivation of *VHL* by germline or somatic mutations causes the constitutive accumulation and activation of HIF-1α and HIF-2α, which induce gene expression programs, alter cellular metabolism, induce angiogenesis, promote metastasis and consequently contribute to the development of clear cell renal cell carcinoma (CCRCC) (Syafruddin 2019). HIF1α also functionally interacts with the NF-κB, producing cytokine TNF-α and the chemokine receptor CXCR4, which are closely associated with the development of human renal-cell carcinoma and other malignant cancers (Vanharanta et al., 2013).

### 2.2 Chronic inflammation affects cancer metastasis

Genomic instability and aberrant proliferative responses caused by chronic inflammation can lead to cancer development, but not the main cause of death in cancer patients (Elinav et al., 2013). Amount of studies have demonstrated that metastasis of tumors is the main cause of death in cancer patients, thus it is vital to investigate the inflammatory mechanisms involved in the metastatic process (Guan 2015). Of note, cancer metastasis requires interactions between tumor cells and immune cells, inflammatory cells or interstitial components.

In response to inflammatory factors (TGF-β, IL-1, and IL-1β) secreted by immune cells, tumor cells undergo EMT (Lee, Kang and Cho 2017; Yang et al., 2020). Subsequently, TAMs typically accumulates around blood vessels and tumor margins and produces various proteases such as urokinase-type fibrinogen activator (uPA) and metalloproteinase 9 (MMP9) that regulate adaptive immunity to promote ECM remodeling, degradation of basement membrane and tumor cell invasion (Mekkawy, Pourgholami and Morris 2014). In addition, CAFs and MDSCs are also recruited to the tumor margin where they exert immunosuppressive effects by interfering with the differentiation of dendritic cells (DCs) (Quail and Joyce 2013).

**FIGURE 2:**
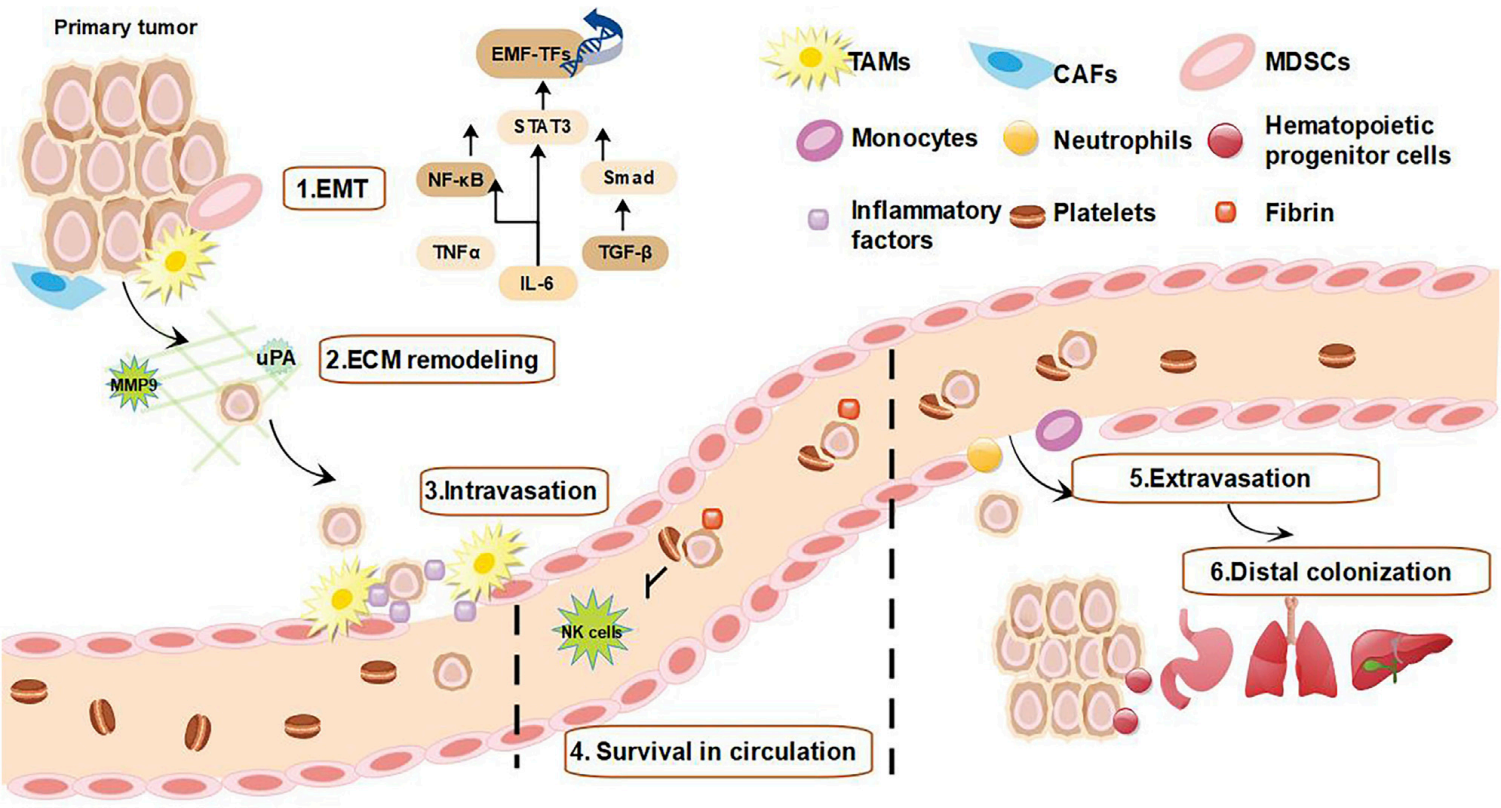
Inflammation promotes tumor metastasis. 1) Inflammatory cells are recruited to the tumor margin and release inflammatory factors, while tumor cells acquire EMT induced by inflammatory factors (Liu and Sun 2021). 2) TAMs and CAFs produce MMP and uPA, causing ECM remodeling (Mekkawy et al., 2014). 3) Tumor cells that acquire EMT interact directly with TAMs to promote tumor cell infiltration (Smith and Kang 2013). 4) Inflammatory mediators increase survival of tumor cells within the circulating metastases by forming protective aggregates with platelets that interfere with NK cell-mediated cytotoxicity and allow tumor cells to reach secondary organs (Kaplan and Jackson 2011; Koupenova et al., 2018). 5) Monocytes and neutrophils promote extravasation of circulating tumor cells by upregulating adhesion factors (Smith and Kang 2013). 6) Finally, tumor cells recruit large numbers of vascular endothelial growth factor-positive hematopoietic progenitor cells to reach metastatic sites, allowing circulating surviving tumor cells to colonize the metastatic site (Vizio et al., 2013; Volk-Draper et al., 2019; Lugano et al., 2020).

Through EMT, the direct reciprocal stimulation between cancer cells and TAMs promotes the infiltration of cancer cells into blood and lymphatic vessels (Smith and Kang 2013). Inflammatory cells produce some mediators such as CXCR4, C-C chemokine receptor 7 (CCR7), and CCR9 which upregulates fibronectin expression and increase the permeability of tumor vessels (Smith and Kang 2013). In addition, inflammatory mediators may increase the survival of circulating metastatic “seeds” (Massagué and Obenauf 2016). Circulating platelets establish protective aggregates with tumor cells that interfere with natural killer cells (NK cells)-mediated cytotoxicity through increasing fibrin deposition and hindering immune cell identification, which allow tumor cells to reach secondary organs (Kaplan and Jackson 2011; Koupenova et al., 2018). Inflammatory cells such as monocytes and neutrophils subsequently upregulate adhesion molecules to promote the extravasation (Hedrick and Malanchi 2022). Tumor-derived C–C motif chemokine ligand 2 (CCL2) binds to CCR2^+^/Ly6C^hi^ endothelial cells to increase vascular permeability and recruit inflammatory monocytes in a p38/MAPK-dependent manner, thereby promoting the extravasation and seeding of metastatic cancer cells (Roblek et al., 2016; Roblek et al., 2019). Finally, cancer cells recruit large numbers of vascular endothelial growth factor (VEGF)-positive hematopoietic progenitors (CD133^+^/CD34^+^/VEGFR3^+^) and colonize distant lateral metastatic sites (Figure 2) (Vizio et al., 2013; Volk-Draper et al., 2019; Lugano et al., 2020).

### 2.3 Chronic inflammation causes cancer therapeutic resistance

Surgery, chemotherapy, radiotherapy and immunotherapy are currently the main options for cancer treatment. However, pro-inflammatory mediators produced by these treatments in turn recruit immunosuppressive cells to reconstitute TME, which further enhances chronic inflammation, subsequently enriches CSCs and induces tumor resistance (Lazzari et al., 2018). CAFs are highly correlated with drug resistance in cancer. Tumor-derived IL-1β and constitutive IL-1 receptor-associated kinase 4 (IRAK4) activate the NF-κB signaling pathway in CAFs and pancreatic cancer cells, thereby attenuating the sensitivity to gemcitabine (Zhang et al., 2018). CAFs-derived IL-6 could upregulate CXCR7 in esophageal squamous cell carcinoma and promote resistance to cisplatin *via* the JAK/STAT3 pathway (Qiao et al., 2018). CAFs promotes EMT and resistance to cisplatin in non-small cell lung cancer (NSCLC) *via* the TGF-β/IL-6 axis; cisplatin treatment also increases the secretion of TGF-β in cancer cells, leading to CAFs activation and increased IL-6 secretion (Shintani et al., 2016). TAMs are also a major cause of drug resistance. Li et al. (2020) found that CCL2 secreted by TAMs increased TAMs recruitment and formed a positive feedback loop with TNF-α secreted from breast cancer cells *via* activating PI3K/Akt/mTOR signaling pathway, promoting tamoxifen resistance in breast cancer cells. IL-6, secreted by TAMs and induces signal transduction and JAK/STAT3, MAPK/ERK or PI3K/AKT activation in tumor, leading to drug resistance (Cao et al., 2015). TAMs are carriers of immune checkpoint ligands, which can interfere with programmed cell death ligand 1(PD-1)/programmed death-ligand 1(PD-L1) and cytotoxic T-lymphocyte antigen 4 (CTLA-4)/CTLA-4 ligand binding by overexpressing PD-L1 or CTLA-4 ligand, and inhibit the immune response of CD8^+^ T lymphocytes and the effect of immunotherapy (Jason and Formenti 2018). MDSCs impede the anticancer activity of immunotherapies including ICT by negatively regulating the expression of immune checkpoint molecules. Higher levels of MDSC numbers are associated with melanoma and other tumors responses to CTLA-4 inhibitors (ipilimumab) (Gabrilovich 2017). In addition, the recruitment of chemokines and cytokines to MDSCs has made MDSCs also resistant to targeted therapies, chemotherapy, and oncolytic viruses-based therapies. Chemokines such as CCL2 cause the resistance of melanoma to BRAF inhibitors by recruiting MDSCs as well (Erin et al., 2020). TGF-β1 secreted by MDSCs increases the sensitivity of NSCLC cells to cisplatin by reversing EMT (Table 2).

**TABLE 2:** Pro-inflammatory mediators causing tumor resistance in the tumor microenvironment (TME).

Pro-inflammatory mediators	Relevant mechanism of action	Related tumor	Related antineoplastic drugs
CAFs	Induce high mobility group box 1	Breast cancer	Doxorubicin Amornsupak et al. (2014)
	STAT3	Ovarian cancer	Cisplatin Zhou et al. (2016)
CAFs (IL-1β)	NF-κB	Pancreatic cancer; non-small cell lung cancer	Gemcitabine Yan et al. (2016)
CAFs (IL-11)	IL-11/Bcl-1	Lung adenocarcinoma	Cisplatin Tao et al. (2016)
CAFs (IL-6)	CXCR7/STAT3/NF-κB pathway	Esophageal squamous cell carcinoma	Cisplatin (Qiao et al. 2018)
TAMs	PI3K-AKT/Survivin	Triple negative breast cancer	Doxorubicin Abd El-Aziz et al. (2022)
	CCL-2/PI3K/AKT/mTOR	Breast cancer	Tamoxifen Li et al. (2020)
	CCL8/JAK1/STAT3	Gastric cancer	5-Fluorouracil He et al. (2021)
	miR-3679-5R/NEDD4L/c-Myc	Lung cancer	Cisplatin (Wang et al. 2021)
	miR-223/PTEN/PI3K/AKT	Ovarian cancer	Cisplatin Zhu et al. (2019)
	CTL activation and proliferation	Breast cancer	Paclitaxel Jason and Formenti (2018)
MDSCs	CCL2/CCR2	Melanoma	BRAF inhibitor PLX4720 Steinberg et al. (2017)
MDSCs (TGF-β1)	ZEB1-driven EMT	EGFR-mutated non-small cell lung cancer	Gefitinib Eser and Jänne (2018)

## 3 Chronic inflammation affects tumor immunity

Mutations, genomic instability and epigenetic modifications can affect the expression of tumor-specific antigen in tumor progenitor cells and activate anti-tumor immunity called “immune surveillance” (Swann and Smyth 2007). During the process, CD8^+^ CTL and CD4^+^ helper T (Th)1 cells prevent cancer development through some mechanisms in which the secretion of interferon (IFN)-γ and cytotoxins are usually involved (Bhat et al., 2017). However, chronic inflammation can also accumulate pro-inflammatory mediators to produce the tumor immunosuppressive microenvironment (TIME). TIME can promote cancer development by promoting cancer cell proliferation, decreasing immunogenicity and evading immune elimination (Garner and de Visser 2020). For instance, IFN-γ activates STAT3 signaling, protects epithelial cells from CD8^+^ T cytotoxic cytokinesis, upregulates the expression of T cell depletion and induces PD-L1 on the transformed epithelial cells recognized by T cells (Zhang et al., 2017). Similarly, inflammatory signals may increase the adaptation and decrease the expression of “stress ligands” on cancer cells, which are required for proper identification. In addition, chronic inflammation and injury trigger the remodeling of tissues, thereby creating space for malignant tumor growth (Mackenzie 2022). After circumventing innate surveillance and establishing a tolerant environment, tumors evade adaptive immune responses in a variety of ways by turning the genetic instability to their advantage (Bhutia, Mallick and Maiti 2010). The mechanisms mainly involve the downregulation of MHC molecules, tumor antigens or the secretion of cytokines such as TGF-β and IL-10 (Levings et al., 2002). Cancer cells also kill aggressive lymphocytes by expressing cell surface molecules that induce apoptosis, such as Fas ligand (FasL) (Zeytun et al., 1997).

## 4 The key players in the tumor microenvironment

### 4.1 Cellular components

It is widely believed that chronic inflammation promotes cancer development, distant metastasis, drug resistance and immunosuppression by providing a tumor-supportive microenvironment termed as the TME (Topalian et al., 2016; Morrison, Byrne and Vonderheide 2018; Lorenzo-Sanz and Muñoz 2019). The TME includes the cancer cells, intrinsic immune cells (TAMs, MDSCs, neutrophils, mast cells, DCs, and NK cells) and adaptive immune cells, as well as surrounding stromal cells (CAFs, endothelial cells, pericytes, mesenchymal cells) (Galli, Borregaard and Wynn 2011). Crosstalk between cancer cells and immune cells ultimately form an environment that prompts cancer development. Recognizing the nature of this dialog may improve therapies and patient outcomes *via* simultaneously targeting multiple components in the TME.

#### 4.1.1 Intrinsic immune cell

##### 4.1.1.1 Monocytes

Monocytes are the main immune cells in TME and are found in almost all tumors with varying degrees of infiltration (Kumar and Gabrilovich 2014). Inflammatory infiltrating monocytes are divided into macrophages and MDSCs. The macrophages infiltrating in the TME are called “tumor-associated macrophages (TAMs)” (Ugel et al., 2015). TAM has two polarization states, namely M1-macrophages (classically activated) and M2-macrophages (alternatively activated) (Shu et al., 2020). M1 macrophages have the positive anti-tumor activity by upregulating the production of cytokines such as IL-6 and IL-1, which activate the T helper type 1 (Th1) reaction and further produce TNF-α, ROS, RNS, and other toxic substances to kill tumors and pathogenic microorganisms (Arora et al., 2018). Macrophages are induced polarization into the M2 type by Th2 cytokines such as IL-4, IL-10, and IL-13. M2 type macrophages are highly endocytic and phagocytic and involved in tissue repair, humoral immunity and metabolism (Atri, Guerfali and Laouini 2018). However, due to TAM is highly heterogeneous in TME, this simplified M1/M2 polarization distinction cannot strictly delineate the phenotypic and functional boundaries of TAMs (Vitale et al., 2019). TAMs appear to share M1 and M2 polarization signatures (Gomes et al., 2013). TAMs affects tumor progression from the following aspects: 1) TAMs produces various factors such as pro-angiogenic factors [e.g., vascular endothelial growth factor A (VEGFA)], TNF, TGF-β, chemokines (CXCL8 and CXCL12) and thymidine phosphorylase, which promote the generation of vascular networks in TME through recruitment and activation of endothelial cells or other cells (e.g., fibroblasts or pericytes) (Cassetta and Pollard 2018). 2) TAMs directly enhances the migration and the aggressiveness of tumor cells through the paracrine loop between macrophages and tumor cells, in which the secretion of colony stimulating factor 1 (CSF1) by tumor cells and epidermal growth factor (EGF) family ligands by macrophages are critically involved (Vitale et al., 2021). In addition, TAMs produce histone proteases and metalloproteinase such as MMP-12, MMP-9, MMP-7, and MMP-2 to remodel ECM and increase immunosuppressive cells infiltration. Then tumor cells could be attracted to the vasculature and come into contact with perivascular tunica interna endothelial cell kinase 2 (TIE2) macrophages that act as conduits for tumor cell escape by expressing VEGFA to increase vascular permeability (B Vendramini-Costa and E carvalho 2012). 3) TAMs mediate immunosuppression and promote immune escape (Binnewies et al., 2018). TAMs suppress the immune microenvironment by secreting chemokines and cytokines such as IL-10, TGF-β, and recruit Tregs to tumor sites to promote cancer development (Li et al., 2021a). TAM agonist homologous ligand protein S (PROS1) secreted by TAMs can bind to TAM receptors MER proto-oncogene, tyrosine kinase (MERTK), AXL and TYRO3, which promote malignant characteristics of tumor cells and promote immune escape by upregulating PD-L1 expression (Burstyn-Cohen, Maimon and Signaling 2019).

##### 4.1.1.2 Myeloid-derived suppressor cells

MDSCs consist of a heterogeneous population of immature myeloid cells (IMC) with potent immunosuppressive activity. Among the major immunosuppressive cells in the TIME, MDSCs are able to activate the NF-κB, STAT3, and promote cancer development (Gabrilovich and Nagaraj 2009). Cancer cells, activated T cells and tumor-associated stromal cells express cytokines such as VEGF, TGF-β, and PGE2, granulocyte-macrophage colony-stimulating factor (GM-CSF) and TNFα, which might cooperatively disrupt myeloid cell maturation, promote the differentiation toward MDSCs, and enhancing MDSCs activation in the TME (Safarzadeh et al., 2018). Subsequently, MDSCs are recruited to tumor sites peripheral and lymphoid organs, then promote cancer development by different mechanisms. MDSCs are demonstrated to remodel TME, regulate EMT process, promote angiogenesis and establish a metastatic ecological niche for cancer dissemination, thereby promoting cancer development and metastasis (Gabrilovich and Nagaraj 2009; Mohme, Riethdorf and Pantel 2017). For instance, MDSCs allow immune escape by inducing incompetence in CD4^+^, CD8^+^ T cells and NK cells. Thus, MDSCs promote immune tolerance to TME and impede the efficacy of cancer immunotherapy (Chesney, Mitchell and Yaddanapudi 2017).

#### 4.1.2 Adaptive immune cells

##### 4.1.2.1 Tregs

Tregs are immunosuppressive subset of T cells identified as CD4^+^ T cells with high CD25/IL-2 receptor α expression. (Hori, Nomura and Sakaguchi 2003). Tregs promote cancer development by exerting suppressive activities on T effector cells (Takeuchi and NishikawEa 2016). In TME, Tregs is thought to be an important mechanism for successful evasion of the immune system in melanoma and head and neck squamous cell carcinoma (Nair, Elkord and Biology 2018). Tregs are highly activated in tumors and characterized by upregulation of the expression of forkhead box protein P3 (FoxP3), immune checkpoints (e.g., CTLA-4, PD-1) and cytokines (e.g., TGF-β) (Saleh and Elkord 2020). Tregs interact with monocytes, B regulatory cells and CAFs in the TME that drives a TIME (Khalaf et al., 2021). For example, Tregs-derived IL-10 alters the macrophages polarization state, causing macrophages to M2-macrophage and exert immunosuppressive effects (Pan et al., 2020). Tregs can inhibit the lytic activity of CD8^+^ through TGF-β, thus effectively suppressing the early tumor-specific immune response of CD8^+^ cells (Chen et al., 2005). Tregs can act on NK cells impairs their effector function by secreting TGF-β (Lazarova and Steinle 2019). Tregs were reported to release granzyme B and perforin and reduce the number of T effector cells in tumors by inducing cytolysis (Liu, Workman and Vignali 2016). In summary, Tregs play an immunosuppressive role in TME, which enhances the infiltration and differentiation of immunosuppressive cells, and limits the activity of cytotoxic NK cells and T cells (Labani-Motlagh, Ashja-Mahdavi and Loskog 2020). Immunosuppressive cells secrete cytokines in response to Treg stimulation, which in turn promote Treg expansion and promote tumor immune escape (Huppert et al., 2022).

### 4.2 Cytokines and other mediator components

In addition to cellular components, TME includes interstitial cells, microvasculature and cytokines that infiltrate into nearby areas, causing the environment for tumor survival under hypoxia, low pH, high interstitial pressure, and fibrosis (Webb et al., 2011; Casazza et al., 2014; Libutti, Tamarkin and Nilubol 2018). In this state, immune/inflammatory cells communicate with cancer cells through secreting large amounts of interleukins, immune checkpoint factors, growth factors, chemokines, colony-stimulating factors, TNF superfamily, and other cytokines to form a local environment conducive to cancer development, angiogenesis and therapeutic resistance.

The inflammatory mediators involve in a complex signaling network coordinated by multiple signaling molecules and transcription factors. Among them, NF-κB and STAT3 signaling pathways are present throughout the TME (Wu et al., 2021). In the canonical NF-κB signaling pathway, excitatory signals activate IL-1 receptors, Toll-like receptors and TNF receptors *via* lipopolysaccharide, IL-1 and TNF-α, respectively. Various bridging proteins and signaling kinases can activate IκB kinase β (IKKβ) in the IKK complex, which in turn degrade IκBα in a phosphorylation-dependent manner. Subsequent transfer of homo- or heterodimers of NF-κB to the nucleus and activation of target gene transcription (Hoesel and Schmid 2013). The target genes include the auto-inhibitory proteins IκBα of negative feedback mechanism, anti-apoptotic genes, adhesion factors, MMP, cyclooxygenase-2 (COX-2), various cytokines and chemokines, thus affecting immunity, chronic inflammation and cell proliferation, apoptosis and metastasis (Oeckinghaus and Ghosh 2009). STAT3 is plays a crucial role in the induction and maintenance of the procarcinogenic inflammatory microenvironment. In the STAT3-related pathway, when cells are stimulated by inflammatory cytokines (IL-6, IL-22, etc.), growth factors [VEGF, EGF, hepatocyte growth factor (HGF), etc.] and other factors (UV, stress and infection), STAT3 is phosphorylated at Tyr 705 residue by JAK, intrinsic receptor tyrosine kinase and several non-receptor tyrosine kinases. The phosphorylation of STAT3, in turn, promotes homodimerization through the interaction between SH2 domain and phosphoryl-Y 705. Then the homodimer transfers to the nucleus and binds to the promoter of target genes, regulating the transcription of target genes (Yu, Pardoll and Jove 2009; Wang et al., 2013). In tumor cells, over-activated STAT3 decreases the expression of immunostimulatory factors, including pro-inflammatory cytokines (IL-12, TNF-α), IFN and chemokines (CCL5, CXCL10), while increasing the expression of certain cytokines (IL-6, TGF-β, and VEGF), thus playing an immunosuppressive effect.

#### 4.2.1 IL-6

IL-6, a multifunctional and pleiotropic cytokine, is secreted by various cells including tumor cells, monocytes, CAFs, adaptive immune cells and vascular endothelial cells (Szekanecz et al., 1994). IL-6 is involved in the regulation of the inflammation, hematopoiesis, immune response, bone metabolism, oncogenesis and even in the “cytokine storm” caused by the novel coronavirus infections (Hirano 2021). High IL-6 levels in the TME reveals the close relationship between chronic inflammation and malignancy (Kumari et al., 2016). IL-6 signaling (mainly *via* the JAK/STAT3 signaling pathway in epithelial and immune cells) can promote chronic inflammation and cancer development (Johnson, O'Keefe and Grandis 2018). IL-6 forms a complex with IL-6 receptor (IL-6R) and gp130, which mediates cancer cell development through the SHP2-ERK-MAPK and STAT3-BCL-2 pathways (Mihara et al., 2012; Heo, Wahler and Suh 2016). Besides, IL-6 can enhance transcriptional induction of molecular targets that affect cell cycle and survival, such as MYC, the apoptosis inhibitor Survivin (Gao et al., 2019). In advanced stages of cancer, IL-6 induces transcriptional activators of EMT (zinc finger proteins Snail1 and Twist), accelerating the metastatic spread of invasive cancer cells (Vu and Datta 2017). Importantly, the growth-enhancing effects of IL-6 on cancer cells also extend to cancer stem cells, capable of self-renewal and expansion, which require STAT3 to act synergistically with stem cell transcription factors such as NANOG (Codd et al., 2018). These interactions ultimately lead to multidrug resistance and progression of multiple tumors, including lung, bladder, breast, ovarian, colorectal and hematological malignancies (Liu et al., 2015; Wei 2019; Manore et al., 2022).

#### 4.2.2 Macrophage migration inhibitory factor

MIFs are secreted by activated T lymphocytes that inhibits monocyte/macrophage migration and maintains macrophage viability. Under physiological conditions, MIF functions as a regulator of inflammatory and immune responses, participating in embryonic development, repairing after tissue trauma and glucocorticoid resistance (Nishihira and Research 2000). Studies have shown that MIF, known as a proto-oncogene, is highly expressed in several tumors such as hepatocellular carcinoma, breast cancer, lung cancer and ovarian cancer, and it is also highly associated with the malignant degree of tumors (Noe and Mitchell 2020; O'Reilly et al., 2016; Mitchell and Yaddanapudi 2014). MIF binds to and activates the receptors CD74/CD44, CXCR4, and CXCR7 by autocrine and paracrine manners (Jankauskas et al., 2019). Upon binding to the receptors, MIF inhibits *p53* expression and activates various signaling pathways (JNK-cJun, PI3K-AKT pathways, etc.) to promote cell proliferation, metastasis, angiogenesis, anti-apoptosis and immune suppression, leading to accelerate cancer development (Lue et al., 2007; Lue et al., 2007; Brock et al., 2014).

#### 4.2.3 Immune checkpoint molecules

Tumor immune escape directly regulates cancer development. Immune checkpoints, including PD-L1/PD-1, CTLA4, lymphocyte activation gene 3 (LAG-3), IDO1 and galectin-9/TIM-3, which is the main mechanism of tumor immune escape *via* inhibiting the activation of effector T lymphocytes (Han, Liu and Li 2020). Among these immune checkpoints, blocking PD-1/PD-L1-mediated tumor immune escape has become a new clinical strategy for tumor therapy.

PD-1 (CD279), a 55 kDa transmembrane protein composed of 288 amino acid residues, is expressed in activated T cells, NK cells and B lymphocytes, monocytes, DCs and macrophages. PD-L1, the first ligand of PD-1, is also widely expressed in activated T cells, B cells, DCs and macrophages. Besides, PD-L1 is found to be highly expressed in cancer cells and therefore considered to be the main factor contributing to tumor immune escape (Yi et al., 2021). Many cytokines (e.g., INF-γ, TNF-α, IL-17, and IL-12) and tumor-derived exosomes in the TME can induce PD-L1 expression and cause tumor immune escape. PD-1 inhibits the activation of T lymphocytes, and in turn, T cell receptor-mediated activation rapidly induces PD-1 expression. PD-L1/PD-1 can be regulated by the following signaling pathways in the TME: PI3K/AKT pathway, NF-κB pathway, WNT pathway, MAPK pathway, JAK/STAT pathway and Hedgehog (Hh) pathway. These signaling pathways enhance tumor immune escape by promoting the expression of PD-1/PD-L1 axis (Jiang et al., 2019b).

## 5 Targeted tumor microenvironment enhances immunotherapy and chemotherapy efficacy

The standard treatments for advanced malignancies, such as chemotherapy and radiotherapy, have made some improvements over the past few decades, but clinical results remain unsatisfactory due to resistance and side effects. The use of immune checkpoint inhibitors for immunotherapy is a promising approach to cancer therapy, although response rates are limited due to the immunosuppressive TME. Emerging evidence supports that optimal therapies should include elimination of tumor cells, disruption of tumor-induced immunosuppression through targeting immunosuppressive cells as well as reactivation of tumor-suppressed effector T cells by checkpoint inhibitors (Table 3).

**TABLE 3:** TME-targeted drugs and immune checkpoint inhibitors (major drugs on the market or in clinical trials).

Target	Related antineoplastic drugs	Related tumors
TAMs Petty et al. (2021)	Imiquimod	Breast cancer (II)
	Motolimod	Squamous cell carcinoma of the head and neck (II)
	PLX3397	Tenosynovial giant cell tumor (III)
MDSCs Li et al. (2021b)	Sargramostim	Melanoma (II)
	Vicriviroc	Colorectal neoplasms (II)
	ATRA	Melanoma (II); Lung cancer (II); Small cell lung cancer (II)
	Epacadostat	Melanoma (III)
MIF Mahalingam et al. (2020)	Imalumab	Colon cancer (I); Non-small cell lung cancer (I); Ovarian cancer (I)
IL-6 (Jean et al., 2015)	Tocilizumab	Non-small cell lung cancer (II); Advanced melanoma (II); Urothelial carcinoma (II)
	Siltuximab	Ovarian cancer (II); Pancreatic cancer (II); Lung cancer (II)
TGF-β Herbertz et al. (2015)	Galunistib	Pancreatic carcinoma (II); Glioblastoma (II); Hepatocellular carcinoma (II)
	Fresolimumab	Breast cancer (II); Renal cell carcinoma (II)
	Vactosertib	Sclerotic fibroma (II)
CTLA Korman, Garrett-Thomson and Lonberg (2022)	Ipilimumab	Unresectable or metastatic melanoma; Adjuvant treatment of melanoma; Advanced renal cell carcinoma; Deficient metastatic colorectal cancer; Hepatocellular carcinoma; Metastatic non-small cell lung cancer; Malignant pleural mesothelioma; Esophageal cancer
PD-1 Markham and Duggan (2018); Marcus et al. (2021); Wolchok et al. (2022)	Pembrolizumab	Melanoma; Non-small cell lung cancer; Head and neck squamous cell cancer; Classical Hodgkin lymphoma; Primary mediastinal large B-cell lymphoma; Urothelial carcinoma; Colorectal cancer; Gastric cancer; Esophageal cancer; Cervical cancer; Hepatocellular carcinoma; Merkel cell carcinoma; Renal cell carcinoma; Endometrial carcinoma; Cutaneous squamous cell carcinoma; Triple negative breast cancer
	Cymplimab	Metastatic cutaneous squamous cell carcinoma
	Nivolumab	Unresectable or metastatic melanoma
PD-L1 Bourhis et al. (2021); Yang et al. (2021); Spigel et al. (2022)	Atezolizumab	Urothelial carcinoma; Non-small cell lung cancer; Small cell lung cancer; Hepatocellular carcinoma; Melanoma
	Avelumab	Metastatic Merkel cell carcinoma; Locally advanced or metastatic urothelial carcinoma
	Durvalumab	Non-small cell lung cancer; Small cell lung cancer

### 5.1 Targeting immunosuppressive cells

#### 5.1.1 Targeting macrophages

TAMs, known as the major infiltrating inflammatory cells in the TME of tumors, contribute to the TIME that promotes cancer development and drug resistance. Therefore, TAMs are attractive targets for tumor therapy aimed at reducing chronic inflammation and TAM-coordinated immunosuppression (Petty and Yang 2017). Several major strategies targeting TAMs have been applied for cancer treatment so far: inhibition of TAMs recruitment (CCL2/CCR2 axis, CCL5/CCR5 axis, etc.), depletion of TAMs [Colony-stimulating factor 1 (CSF1)/CSF1R axis, etc.], induction of M1 macrophage polarization [CD47/Signal regulatory protein alpha (SIRPα) axis, CD40/CD40L, etc.] and enhancement of macrophage-mediated phagocytosis [PD-1/PD-L1axis, MHC-1/leukocyte immunoglobulin like receptor B1 (LILRB1), etc.] (Anfray et al., 2019; Zhou, Liu and Huang 2021b). A number of preclinical and clinical studies with small molecule inhibitors and antibodies (such as PF-04136309, Maraviroc, BLZ945, Hu5F9 -G4, Duvelisib, FPA008, etc.) have shown that the TAM-targeted therapy is an effective anti-tumor strategy (Cassetta and Pollard 2018). For example, PF-04136309, a small molecule CCR2 inhibitor, inhibits tumor-associated macrophage infiltration and enhances endogenous anti-tumor immunity, and has performed well in clinical studies for advanced pancreatic cancer (Nywening et al., 2016). Additionally, some drugs have been reported to induce differentiation of macrophages to a pro-inflammatory phenotype and inhibit tumor progression, such as Imiquimod, the only clinically approved toll-like receptor (TLR) ligand, exerts anti-tumor activity in basal cell carcinoma, melanoma and breast cancer (Qiu et al., 2018).

#### 5.1.2 Targeting myeloid-derived suppressor cells

MDSCs, main components of the immunosuppressive TME, which induce unresponsiveness of NK cell, CD4^+^ and CD8^+^ T cell, thereby promoting immune tolerance to tumors. The expand of MDSCs has been reported to be associated with cancer development, therapeutic resistance, reduced efficacy of immunotherapy and poor prognosis of patients. Thus, MDSCs are considered as promising targets for cancer therapy. The main strategies to target MDSCs include: 1) depletion of circulating and tumor-infiltrating MDSCs [blocking VEGF and receptor tyrosine kinase (c-Kit) signaling; anti-CD33]; 2) prevention of MDSC recruitment and trafficking (CCL3, CCL4, CCL5-CCR5; CCL2, CCL5-CXCR2; CSF-1R/CSF-1); 3) Inhibition of MDSC immunosuppressive function [disruption of COX-2/PGE2 signaling; activation of nuclear factor erythroid 2-related factor 2 (Nrf2)]; 4) Differentiation of MDSCs into a non-suppressive immune state (promotion of IMC differentiation; epigenetic reprogramming) (Law, Valdes-Mora and Gallego-Ortega 2020). Currently, small molecule inhibitors and antibodies targeting the above-mentioned strategies (cabazitaxel, bevacizumab, rapalisin, paclitaxel, leronlimab + carboplatin, tadalafil, omadacyclovir, GTB-3550 TriKE™, etc.) have achieved good results in phase II clinical trials (Li et al., 2021b). For instance, MDSCs express S100A8/A9 and the receptor for advanced glycation endproducts (RAGE), which in turn recruit MDSCs and enhance their immunosuppressive function constituting positive feedback loop. Tasquinimod, an oral S100A9 inhibitor, that causes depletion of blood mononuclear cells, suppression of MDSCs recruitment and inhibition of metastasis, has been demonstrated to improve progression-free survival in patients with metastatic debulking-resistant prostate cancer (Li et al., 2021c).

#### 5.1.3 Targeting T cells

T cell-specific cancer immunotherapies are currently available to release the antitumor efficacy of T cells by suppressing immune checkpoints or to enhance adaptive immunity by using genetically engineered T cells equipped with chimeric antigen receptors (CARs) or T cell receptors (Waldman, Fritz and Lenardo 2020). In recent years, immune checkpoint inhibitors with CTLA-4, PD-1 and PD-L1 monoclonal antibodies has become a successful treatment for several advanced cancers (Wojtukiewicz et al., 2021). To date, there are seven immune checkpoint inhibitors have been approved by U.S. food and drug administration (FDA) for certain cancer treatment (melanoma, NSCLC, lymphoma, urothelial carcinoma, etc.), including CTLA-4 inhibitors (Ipilimumab), PD-1 inhibitors (Pembrolizumab, Nivolumab, Cymplimab), PD-L1 inhibitors (Atezolizumab, Avelumab and Durvalumab) (Naimi et al., 2022). In addition, the combination of immune checkpoint inhibitors, such as Ipilimumab and Nivolumab, has recently been approved for unresectable malignant pleural mesothelioma, hepatocellular carcinoma, metastatic NSCLC, advanced renal cell carcinoma, esophageal squamous cell carcinoma, metastatic colorectal cancer and melanoma (Chen et al., 2020). Even so, the approved indications for immune checkpoint inhibitors are expected to increase, and several new agents are under investigation in clinical trials.

### 5.2 Targeting cytokines in the tumor microenvironment

Cytokines such as MIF, IL-6, and TGF-β used as adjuvants in cancer treatment has been extensively investigated in many clinical studies. The current therapeutic strategy targeting MIF is anti-MIF monoclonal antibody. Imalumab, an anti-MIF monoclonal antibody, is the only candidate that has progressed and has been well tolerated in clinical trials for the treatment of solid tumors (O'Reilly et al., 2016). IL-6 inhibitors include Tocilizumab, an anti-IL-6R monoclonal antibody, and Siltuximab, an anti-IL-6 monoclonal antibody (Johnson et al., 2018). Tocilizumab overcomes chemoresistance in mesenchymal stem cell-like breast cancer (Chung et al., 2022). Currently, drugs targeting TGF-β in clinical trials contains neutralizing antibodies, small molecule inhibitors and so on. For example, the TGF-β chelator GC1008 (also known as Fresolimumab) is one of the most characterized monoclonal anti-TGF-β1-3 antibodies, showing an acceptable safety and antitumor activity for patients with malignant melanoma or renal carcinoma in a phase one clinical trial (Morris et al., 2014; Liu, Ren and ten Dijke 2021). Vactosertib has a good suppressive effect on sclerotic fibroma as a potential small-molecule TGF-β type I receptor kinase inhibitor (Kim et al., 2021).

### 5.3 Combination of tumor microenvironment-targeted agents with immunotherapy or chemotherapy overcome tumor resistance

Studies have showed that chemotherapy or immunotherapy alone promotes immunosuppression of tumors through TME, resulting in drug resistance and treatment failure. Thus, a growing number of preclinical and clinical trials have focused on the combination of TME-targeted agents with chemotherapy or immunotherapy (Zhu et al., 2021). Recently, TME-targeted agents combined with immunotherapy in clinical studies has demonstrated new possibilities for long-term tumor control in patients. It is a promising therapeutic strategy by the combined inhibition of immune checkpoint and TGF-β signaling pathways because these critical pathways have independent and complementary immunosuppressive functions. Vactosertib in combination with Durvalumab demonstrates safety and efficacy in patients with metastatic NSCLC and urothelial carcinoma (Kim et al., 2021). Liver X receptors β (lxRβ) agonist RGX-104 induces depletion of immunosuppressive MDSCs (granulocytes and monocytes). Phase I clinical trials are ongoing to evaluate the effectiveness of the combination of RGX-104 and immune checkpoint inhibitors such as Nivolumab or Ipilimumab in patients with lymphoma (Murciano-Goroff, Warner and Wolchok 2020). Galunisertib, a heterotetrameric complex of paired type I (TβRI) kinase inhibitor, has been used in combination with multiple chemotherapies (such as paclitaxel, gemcitabine, sorafenib, etc.), which can improve anti-tumor efficacy of patients with triple-negative breast cancer, glioblastoma and pancreatic ductal adenocarcinoma (Liu et al., 2021).

## 6 Future outlooks

Increasing evidences show that chronic inflammation can influence all aspects of cancer development along with the response to therapy. TME consisting of innate and adaptive immune cells, pro-inflammatory cytokines and chemokines accompanies the entire phase of cancer development and drug resistance. Under normal conditions, the immune system identifies and eliminates both pathogens and tumor cells to inhibit tumor growth. However, inflammatory cells and cytokines may contribute to cell survival, proliferation, invasion and angiogenesis as tumor promoters during chronic inflammation. Chronic inflammation also induces cancer development by altering the TME and important signaling pathways such as NF-κB and STAT. Oncogenic alterations can enable tumors to promote an inflammatory environment, which benefits tumors. Therefore, therapies targeting chronic inflammation will add new weapons to the arsenal of cancer therapy. In terms of therapeutic interventions, TME an impact on cancer development, immunosuppression and drug resistance that make it an attractive target for sensitizing tumors to conventional therapies as well as an alternative option for drug-resistant tumors. Targeting inflammatory infiltrating cells and cytokines in the TME is a potentially useful adjunct to immunotherapy and conventional treatment of tumors. However, TME and key signaling pathways vary widely between tumor types and tissues, how to manage the diversity and response to cancer treatment infecting by different TME will be an important area for future research.

While immune checkpoint therapy has a positive therapeutic effect and potential cure for a small number of patients with advanced high-grade tumors, TME evolved dynamically through a compensatory feedback mechanism blocks the effect of immunotherapy, generates drug resistance and cancer development. To address this limitation of immunotherapy, we can try to design multi-targeted immunotherapy strategies and develop new integrated immune activation strategies. It is also possible to modulate TME to kill cancer with autoimmune potential and to reduce the possible adverse effects of specific targeted therapies.
